# Understanding Medical Students’ Perceptions of and Behavioral Intentions toward Learning Artificial Intelligence: A Survey Study

**DOI:** 10.3390/ijerph19148733

**Published:** 2022-07-18

**Authors:** Xin Li, Michael Yi-chao Jiang, Morris Siu-yung Jong, Xinping Zhang, Ching-sing Chai

**Affiliations:** 1 Department of Infectious Disease, The First Affiliated Hospital of China Medial University, Shenyang 110000, China; lixin2021@cmu.edu.cn; 2 Department of Curriculum and Instruction, Faculty of Education, The Chinese University of Hong Kong, Hong Kong SAR, China; mjiang@cuhk.edu.hk (M.Y.-c.J.); mjong@cuhk.edu.hk (M.S.-y.J.); cschai@cuhk.edu.hk (C.-s.C.); 3 Centre for Learning Sciences and Technologies, Hong Kong Institute of Educational Research, The Chinese University of Hong Kong, Hong Kong SAR, China; 4 Hunnan Tumour Centre, The First Affiliated Hospital of China Medical University, Shenyang 110000, China

**Keywords:** artificial intelligence, medical students, behavioral intention, theory of planned behavior

## Abstract

Medical students learning to use artificial intelligence for medical practices is likely to enhance medical services. However, studies in this area have been lacking. The present study investigated medical students’ perceptions of and behavioral intentions toward learning artificial intelligence (AI) in clinical practice based on the theory of planned behavior (TPB). A sum of 274 Year-5 undergraduates and master’s and doctoral postgraduates participated in the online survey. Six constructs were measured, including (1) personal relevance (PR) of medical AI, (2) subjective norm (SN) related to learning medical AI, (3) perceived self-efficacy (PSE) of learning medical AI, (4) basic knowledge (BKn) of medical AI, (5) behavioral intention (BI) toward learning medical AI and (6) actual learning (AL) of medical AI. Confirmatory factor analysis and structural equation modelling were employed to analyze the data. The results showed that the proposed model had a good model fit and the theoretical hypotheses in relation to the TPB were mostly confirmed. Specifically, (a) BI had a significantly strong and positive impact on AL; (b) BI was significantly predicted by PR, SN and PSE, whilst BKn did not have a direct effect on BI; (c) PR was significantly and positively predicted by SN and PSE, but BKn failed to predict PR; (d) both SN and BKn had significant and positive impact on PSE, and BKn had a significantly positive effect on SN. Discussion was conducted regarding the proposed model, and new insights were provided for researchers and practitioners in medical education.

## 1. Introduction

Artificial intelligence (AI) is developing rapidly with faster computing, advancement in hardware (e.g., fifth-generation mobile networks) and the advent of deep learning and artificial neural networks [1]. AI is found to be broadly benefitting modern medicine and clinical practice [2]. A range of AI-based applications, such as clinical and genomic diagnostics [3,4], chronic disease management [5], blockchain technology [6] and sophisticated surgical robots [7] have deeply reshaped medical practice in the 21st century. As an emerging field, AI will only continue to transform the way medical science advances and become an integral part of medicine in the future [8]. Although it has been pointed out that AI will be one of the main elements of medical education in the upcoming decade [9], research is lacking with respect to factors that may impact medical students’ intentions to adopt AI technologies [10], and little is known about medical students’ attitude toward the use of AI in clinical practice [11]. Therefore, empirical studies on medical students’ perception of and their behavioral intention (BI) toward AI are needed.

Moreover, research findings are inconsistent regarding the “intention-behavior” gap in relation to technology adoption [12]. Very little research has been conducted to examine medical students’ intention to learn medicine-related AI and their actual learning behavior. Hence, the impact of AI on medical education and training remains unclear [13]. On the other hand, medical students are also facing the alleged challenges brought by the rapid development of AI and have concerns of being replaced by future AI [14]. Such concern may complicate how medical students perceive AI for medical treatment. For example, there is research pinpointing that AI might reduce educational opportunities to develop “clinical judgement” and “practical skills” of trainee doctors [13]. Since AI applications will re-configure the roles of human practitioner and machines in medical care, medical educators and stakeholders need to understand medical students’ intention to learn AI in clinical practice and their actual learning to surface possible gaps and bridge the gaps. Ideally, medical students need to possess adequate knowledge about medical AI and embrace the use of medical AI. This will help them to use the technology critically and contribute to the development of medical AI in service of patient care. Continuous intention to learn about useful technology is dependent on a number of interrelated factors that vary in different contexts.

Against this backdrop, the current study investigated medical students’ perceptions of AI, focusing specifically on their intention to learn medical AI and its influential factors. To this end, the theory of planned behavior (TPB) [15] was adopted in this study as the theoretical framework, based on which a questionnaire was developed and validated by revising measurement instruments employed in previous studies. Students’ attitudinal beliefs, normative beliefs and perceived control beliefs were evaluated, and the hypotheses regarding the relationships between those beliefs and students’ BI were tested.

## 2. Theory and Hypotheses

### 2.1. Theory of Planned Behavior

**Figure 1:**
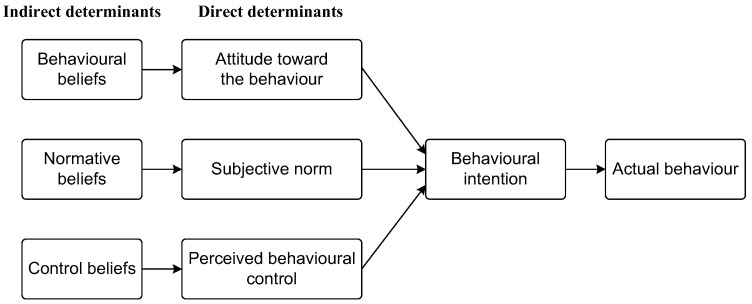
Framework of the TPB (Adapted from [31]).

The TPB is an explanatory model that has been widely applied to the prediction of and changes in behavior [16] in a variety of behavioral domains. It postulates that volitional human behavior is immediately predicted by BI to engage in this behavior, and in turn, BI is determined by three direct factors, namely, attitude toward the behavior, subjective norm (SN) regarding the behavior and perceived behavioral control (PBC) [15]. These three factors fall under behavioral beliefs, normative beliefs and control beliefs, respectively (Figure 1). As stated by Ajzen [16] (p. 315), “a favorable attitude and a supportive subjective norm provide the motivation to engage in the behavior but a concrete intention to do so is formed only when perceived control over the behavior is sufficiently strong”. According to a range of synthesis works with respect to the TPB (for syntheses of some of this research, see [17,18,19,20,21]), those three factors have unfailingly accounted for the variances in BI in various contexts such as choice of travel mode [22], environmental protection [23,24], healthcare [25,26], educational studies [27,28] and technology adoption [29,30].

On the other hand, some researchers have made attempts to extend or revise the TPB to probe into context-specific issues [32]. For example, Kang et al. contextualized the TPB in a study examining athletes’ deviant behaviors by adding ethical obligation as a fourth predictor of BI [33]. In addition, the revised TPB in the study was further extended by measuring athletes’ actual deviant behaviors (i.e., frequency of their engagement in deviant behaviors such as rule-bending and violence). Within the domain of technology use in education, there may exist particular drivers and inhibitors of intention to adopt technology. In studying Singaporean teachers’ technology use, Teo et al. also extended the TPB by adding four external variables (i.e., perceived usefulness, perceived ease of use, management expectation and technical support) as precedents of the three belief-related endogenous variables (i.e., attitude, SN, and PBC) in the original TPB [34]. The extended TPB was reported to have good model fit, and 71.7% of the variances in BI were accounted for. All the hypotheses were supported, except for two pertaining to SN. As was argued by Ajzen [15,16], the inclusion of additional variables is acceptable providing the justification is theoretically strong and a significant portion of variance in intentions or behavior is captured. In the context of the current study, according to the TPB, students’ actual learning (AL) of medical AI technologies will be predicted by their BI toward the learning behavior. Thus, hypothesis H1 is formulated below:

**H1.**  *BI toward learning medical AI technologies predicts medical students’ AL of the technologies.*

### 2.2. Attitude toward Learning Medical AI

As expectancy-value formulation is foundational to the formation of attitude toward a behavior [16], the TPB defines attitudes toward behavior as an individual’s expectations and experience of the consequences of performing a certain behavior [35]. It posits that attitudes toward the behavior are developed and revised according to assessments about beliefs and values [36]. The adoption of a given technology is generally based on users’ intrinsic involvement, which is further based on the personal relevance (PR), psychological significance and significant consequences that the technology has for them [37]. Therefore, research pertaining to technology adoption commonly considers PR as a sufficient condition for users’ positive attitude toward the use of a given technology [38], and the relevance of a given technology is theoretically operationalized as an antecedent of perceived usefulness [37,39].

Correspondingly, in the context of the current study, attitude toward learning medical AI is shaped by the medical students’ PR of AI in clinical practice. Medical students who understand AI technology as more relevant in their clinical practice may have more positive expectations and experiences of medical AI and thus hold a more positive attitude toward medical AI. PR of medical AI technologies measures how the AI technologies are able to enhance medical students’ productivity or performance. In literature pertaining to technology adoption, a plethora of primary studies have obtained positive evidence of the positive impact of attitude on behavioral intention [40,41]. Because it is considered a factor which behavioral studies commonly employ as an estimate of attitude toward behavior, PR is oftentimes considered as an antecedent of BI [42]. Accordingly, in the current study, it is hypothesized that:

**H2.**  *PR of medical AI technologies predicts medical students’ BI to use the technologies.*

### 2.3. Subjective Norm of Learning Medical AI

SN, or social influence, refers to an individual’s perception of social pressure from important others to engage in the certain behavior in question [22]. It is based on accessible normative beliefs and acts as a contextual source of influence on behavior [16]. Normative beliefs can be injunctive or descriptive, and either type contributes to SN to engage in a given behavior [43]. An injunctive normative belief is the expectation or subjective estimation that important others approve or disapprove of performing the behavior in question. In contrast, descriptive normative beliefs are beliefs about whether important others perform the behavior themselves [16]. When important others believe that a certain behavior benefits the individual or perform the behavior themselves, the individual may be more willing to engage in the behavior under consideration [44] and vice versa. In the context of this study, the important others can be peers and course teachers in medical institutions or other doctors in hospitals. When these referents believe that learning medical AI is beneficial in clinical practice, a medical student may be encouraged and more willing to engage in behaviors associated with learning medical AI. Conversely, a medical student may be discouraged to learn about medical AI if their referents believe they need not or should not learn it.

Extant studies have consistently found that a supportive SN has significant and positive effect on BI related to technology adoption, and such an effect was either direct or indirect. Rajeh et al. identified factors that influenced students’ satisfaction and continued intention toward e-learning under the influence of COVID-19 [45]. Their results based on the TPB revealed that, among other factors, SN had a significant and positive influence on their intention to use technology. To and Tang explored the antecedents of college students’ intention to participate in computer-based course evaluation and uncovered that SN had direct effect on students’ BI [46]. On the other hand, they also found that SN had a positive, strong and significant effect on PR, which in turn predicted perceived ease of use and perceived usefulness. Likewise, numerous studies discovered that attitudinal variables such as PR in a range of technology acceptance models mediated the effect of SN on BI. For example, Teo revealed that SN had significant but indirect effect on BI of technology adoption, and the effect was mediated by attitude toward the behavior [47]. Jiang et al. validated an extended technology acceptance model against the background of COVID-19 and observed that SN was the only external factor that had a significant effect on BI to use technology through both perceived usefulness and perceived ease of use [40]. Based on the above, hypotheses H3a (in tandem with H2 aforesaid) and H3b are formed with regard to the indirect and direct effects of SN on BI, respectively.

**H3a.**  *SN related to learning medical AI technologies predicts medical students’ PR of the technologies.*

**H3b.**  *SN related to learning medical AI technologies predicts medical students’ BI to use the technologies.*

### 2.4. Perceived Behavioral Control over Learning Medical AI

PBC is defined as one’s perceived capability of performing a behavior [15], and it is conceptually similar to Bandura’s perceived self-efficacy (PSE) [30,48]. Following Ajzen [16], there is little conceptual difference between PBC and PSE, because both terms indicate volitional human beliefs about their capability of performing a given behavior. Although operationally the perspectives of measuring PBC and PSE are usually different [16], in practice, empirical studies commonly adopt PSE as an estimate of PBC [30]. In various contexts, research pertaining to the TPB obtained supportive findings that PBC (operationalized as PSE) is positively associated with BI [22,33]. In turn, self-efficacy is developed partially either based on vicarious experiences individuals undergo when they observe others performing similar tasks or as a result of social persuasions or verbal messages they receive from others [49]. Accordingly, SN is considered as a major source of self-efficacy, in reference to which empirical studies obtained confirmative evidence [50,51,52]. Thus, it is posited that:

**H3c.**  *SN related to learning medical AI technologies predicts medical students’ PSE of learning the technologies.*

Additionally, studies in numerous contexts have demonstrated that PSE is a good predictor of attitudinal factors [53,54], indicating a possible indirect effect of PSE on BI through attitudinal factors. In particular, self-efficacy is commonly integrated into different extended technology acceptance models as a significant external variable in primary studies [55,56], and review studies in this domain also concluded that PSE had positive impact on attitude toward technology adoption (for synthesis studies see [57,58]). Although there are studies pointing out that the direction of this relation (i.e., self-efficacy→attitude) is not deterministic, as positive attitudinal factors may also enhance self-efficacy [59], but in the domain of technology adoption, most studies claimed that users’ self-efficacy beliefs predicted their attitude toward their technology use. Thus, hypotheses H4a and H4b are formed:

**H4a.**  *PSE of learning medical AI technologies predicts medical students’ PR of learning the technologies.*

**H4b.**  *PSE of learning medical AI technologies predicts medical students’ BI to learn the technologies.*

### 2.5. Medical AI Literacy

Apart from the three direct determinants (Figure 1), background factors also contribute to the prediction and change in BI toward the behavior [43], and those factors can be individual (e.g., personal traits), social (e.g., education history) or epistemic (e.g., knowledge and ways of thinking). Following Chai et al. [30], AI literacy is fundamental to the behavioral, normative and control beliefs that would determine BI toward learning AI technologies. Based on Moore’s definition of technology literacy [60], in the context of learning medical AI, an individual is medically AI literate if they know how medical AI technologies work and how to use those technologies to solve clinical problems, such as using AI-based applications to acquire, interpret and apply knowledge in clinical practice. As evident, students’ basic knowledge (BKn) of medical AI technologies will constitute their medical AI literacy, i.e., knowing at least conceptually how medical AI technologies work and what are the problems that such technologies can address [32]. Their BKn of medical AI technologies may play a critical role in predicting their BI toward learning medical AI technologies. Previous studies have demonstrated that perceived technology or digital literacy predicts teachers’ and students’ BI to engage in mobile learning [61,62]. Consequently, hypothesis H5a is formulated:

**H5c.**  *BKn of medical AI technologies predicts medical students’ BI to learn the technologies.*

Chai et al. [30] argued that AI literacy is “foundational to the behavioral, normative and control beliefs that would consequently predict the BI”, (p. 91) indicating that apart from the possible effect on BI, BKn of medical AI technologies may be a predictor of the three determinants of BI, i.e., PR, SN and PSE. Firstly, it is obvious that the perception of social pressure from important others (i.e., SN) to engage in technology adoption will depend on the individual’s technology or digital literacy. To be specific, when an individual is medically AI literate, it is less likely for him or her to perceive much pressure from important others to adopt AI technologies in medical practice. Conversely, if an individual is not medically AI literate, he or she may receive more social pressure from important others in applying AI technologies in clinical practice. Second, in terms of the relationship between BKn and PSE, as noted by Bandura [49], one source of PSE is one’s own enactive mastery experience, i.e., the interpreted results of one’s own performance. Therefore, operationally, acquired BKn of medical AI technologies is assumed to contribute to students’ PSE of learning medical AI technologies. Empirical studies examining the relationship between AI literacy and PSE demonstrated that AI literacy was a good predictor of PSE in learning AI [30]. Third, studies in medical education revealed that basic science knowledge was perceived by medical students as contributory to the development of adaptive expertise and professional identity formation [63]. For medical students to make competent clinical decisions assisted by AI-based applications, they must be medically AI literate and retain AI-related knowledge from the preclinical phase of their medical course [64]. Correspondingly, it can be assumed that medical students’ knowledge of AI technologies may contribute to the practical applicability of the AI technologies to the clinical setting, and their BKn may also be a predictor of their PR of learning medical AI technologies. Based on the foundational role of BKn in learning medical AI technologies, it is posited that:

**H5a.**  *BKn of medical AI technologies predicts medical students’ SN related to learning the technologies.*

**H5b.**  *BKn of medical AI technologies predicts medical students’ PR of learning the technologies.*

**H5d.**  *BKn of medical AI technologies predicts medical students’ PSE of learning the technologies.*

**Figure 2:**
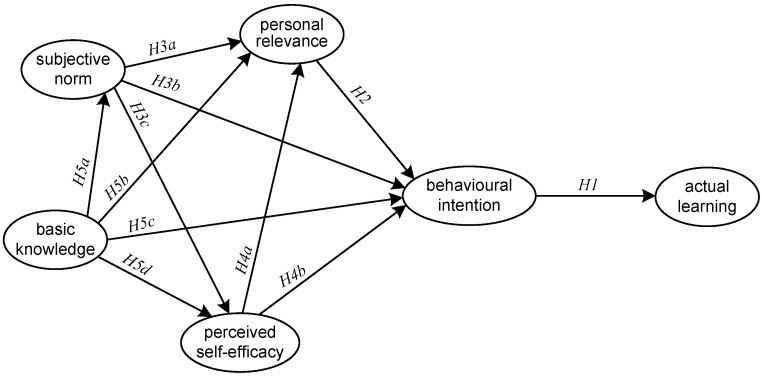
Proposed structural model based on the TPB.

To conclude, based on the findings from previous studies with respect to the interplay among the variables (i.e., PR, SN, PSE and BKn) in the TPB, the present study proposed a structural model to test the corresponding hypotheses formulated for medical students in the context of AI applications (Figure 2).

## 3. Method

### 3.1. Participants

The current study recruited senior medical students (Year-5 undergraduates and postgraduates) who had had some preclinical experience of working as trainee doctors because they had more opportunities to be exposed to clinical practices involving medical AI than their junior counterparts. A sum of 274 undergraduates in Year 5 and master and doctoral postgraduates from one medical university in Chinese mainland participated in the online questionnaire survey. The undergraduates majored in clinical medicine and nursing, and the postgraduates were from a range of medical majors such as surgery, urology, intensive care, gynecology and obstetrics, pediatrics, public health, dermatology, and clinical pharmacy, etc. Their average age was 23.8 years old; 29.9% of them were male and 70.1% female; 17.9% had experience of working as doctors in public hospitals. In terms of their self-reported information and communications technology (ICT) skills, 83.9% reported that they were competent in using office software and understood the technical parameters of commonly used electronic hardware. Another 12.4% reported that they could use specialized medical software to deal with clinical and research-related issues. Only 3.6% reported that they were able to build up a local area network or write computer programs using at least one programming language. The study was approved by the university, and the participants were well-informed of the purpose of the survey and gave their consent before participating in the survey.

### 3.2. Measures and Instruments

The first part of the questionnaire covered the background and demographic data, including gender, age, program level, work experience, and ICT skills. The second part of the questionnaire was composed of 25 items which measured the six constructs in the present study, namely, (1) personal relevance of medical AI, (2) subjective norm related to learning medical AI, (3) self-efficacy in learning medical AI, (4) basic knowledge of medical AI, (5) behavioral intention to learn medical AI and (6) actual learning of medical AI. Except for the self-constructed measure of actual learning of medical AI, the items of the other five measures were all adapted from previous studies pertaining to AI learning and purposefully contextualized into the medical setting. Each item was a statement which was scored on a 6-point Likert scale from 1 = “*not true of me at all*” to 6 = “*very much true of me*”. The items were finalized after a pilot study and presented in the Appendix A.

**Personal relevance of medical AI** was redeveloped with reference to Chai et al.’s questionnaire regarding behavioral intention to learn AI [32] and validated through confirmative factor analysis (CFA) in this study. The four items measured participants’ perceived usefulness in understanding fundamental concepts pertaining to medical AI and applying related skills in clinical practice. As aforementioned, perceived usefulness of AI is conceptually similar to personal relevance of AI, as both constructs measure the practical applicability of the AI technology to the contextualized setting.

**Subjective norm related to learning medical AI** was composed of four items which were also redeveloped from Chai et al.’s work [32] and validated via CFA in the current study. The items indicated how the participants’ peers, course teachers, mentors and stakeholders (e.g., medical institution) thought of learning AI technologies used in medical practice, and measured participants’ both injunctive and descriptive normative perceptions of learning medical AI.

**Self-efficacy in learning medical AI** was revised from Chai et al.’s questionnaire concerning students’ self-efficacy in learning AI [30] and was validated via CFA in this study. This scale was composed of four items, which covered a graded series of potential obstacles to learning medical AI (from understanding fundamental concepts to applying skills related to medical AI) and asked the participants to indicate how likely it was that they could overcome each obstacle [16]. According to Fishbein and Ajzen [43], greater confidence suggested greater self-efficacy, and those who scored higher on this dimension might have stronger self-efficacy in using AI technologies in clinical practice.

**Basic knowledge of medical AI** was redeveloped based on Chiu et al.’s survey [65] and Chai et al.’s survey [32] and validated through CFA, both of which measured respondents’ understanding of their basic knowledge regarding AI. The original questionnaires covered six items and four items that measured AI literacy and basic knowledge of AI, and both had satisfactory internal reliability. In the current study, five items were adapted from the two surveys and were purposefully contextualized for measuring basic knowledge of medical AI.

**Behavioral intention to learn medical AI** was also revised from Chai et al.’s questionnaire [32] and validated via CFA, which was composed of four items measuring participants’ behavioral intention to use and learn medical AI technologies. The adaptation was also a contextualization of the items toward the specific learning and use of medical AI.

**Actual learning of medical AI** was self-constructed and validated through CFA in this study. It was composed of four items in the present study which measured the participants’ actual behavior associated with medical AI learning. To be specific, the items asked the participants to reflect on their experience of learning and practicing with medical AI technologies, including online and offline self-learning, with electronic and paper-based materials, in a lecture-based and hands-on form.

### 3.3. Data Collection and Analysis

A pilot survey on 34 participants was conducted before the final administration of the questionnaire. The participants in the pilot were excluded from the final sample. Item analysis was conducted to calculate item discrimination, item-total correlation and internal reliability (estimated by Cronbach’s α) based on the pilot results. The pilot revealed that one PSE item and one BKn item were not psychometrically acceptable because of their low item discrimination (i.e., non-significant difference between high- and low-performers). In tandem with the feedback from the pilot participants, we decided to remove those two items from the finalized survey, leaving 23 items in the finalized questionnaire.

Before the administration of the finalized survey, three additional “filtering items” were purposefully added in order to filter out possible “careless respondents”. Each filtering item was a semantically equivalent item to the original item in the questionnaire and thus formed three pairs of synonymic statements. Two professors in the field of educational technology were consulted to ensure the surface validity of the three pairs. Following Jiang et al.’s method [40], a filtering criterion was applied in the data preprocessing stage to exclude those whose responding performance was inconsistent. To be exact, if the sum of the absolute value of the averaged difference between all the three filtering pairs was greater than 1 unit per pair, then the responding performance of the participant was defined as inconsistent, and thus the corresponding data record was considered invalid for further analysis and should be removed from the sample. Afterwards, manual scrutiny of the responses was carried out, and responses of a “specific” pattern (e.g., straight 4′s, 111222333444…, 123412341234…) were also identified for exclusion. Accordingly, a sum of 63 records were removed from the sample, leaving 211 cases for further analyses.

CFA was first performed to examine the construct validity of the proposed model, and then the structural equation modelling (SEM) was undertaken estimating all path coefficients. Specifically, this study adopted Chi-square (*χ*^2^), degree of freedom (*df*) in tandem with their significance values (*p*) and other model fit indices, including the comparative fit index (CFI), the Tucker–Lewis index (TLI), the root mean square error of approximation (RMSEA) and the standardized root mean square residual (SRMR) to evaluate the model fit. According to Huang et al. [66], the model fit is good when *χ*^2^/*df* is <3 and sometimes permissible when <5. Furthermore, CFI and TLI should be ≥0.95 for an excellent model fit and ≥0.90 for an acceptable model fit [67]. RMSEA and SRMR should be <0.06 and 0.08, respectively, for an excellent model fit, and 0.08 and 0.10, respectively for an acceptable model fit [68].

## 4. Results

### 4.1. Construct Validation

Before investigating the structural relationships in the proposed model, CFA was conducted to validate the constructs of the six constructs. The results showed that the measurement model had a good fit (*χ*^2^ = 490.388, *df* = 215, *χ*^2^/*df* = 2.281, *p* < 0.001, CFI = 0.950, TLI = 0.941, RMSEA = 0.078, and SRMR = 0.044). Descriptive statistics showed no floor or ceiling effect according to the means calculated at the item level (Table 1).

Table 2 shows the correlation matrix between the six constructs estimated by Pearson product–moment correlation coefficient. The descriptive statistics demonstrated no floor or ceiling effect on the construct level. Normality testing revealed that except for BI, the data of the other five constructs were all normally distributed. The magnitude of their skewness ranged from 0.06 to 0.91, less than the threshold of 1 [69], and the magnitude of their kurtosis ranged from 0.02 to 1.18, less than the threshold of 2.20 [70]. As for BI, the magnitude of its skewness is 1.07, and the magnitude of its kurtosis 2.39, which only marginally exceeded the suggested thresholds, respectively. Therefore, BI could be assumed to have a roughly normal distribution. The Cronbach α values ranged from 0.85 to 0.98, indicating that the measurement was of fine internal reliability.

Furthermore, the construct reliability, convergent validity and discriminant validity were examined, and the results in Table 3 indicated that the composite reliability (CR) ranged from 0.86 to 0.98, and McDonald’s construct reliability (MaxR(H)) ranged from 0.87 to 0.99, achieving fine construct reliability (>0.7) in the proposed model [71]. In addition, the values of average variance extracted (AVE) of all the six constructs were above 0.5, establishing good convergent validity of the measurement model on the construct level [69]. In addition, except for SN, the values of maximum shared variance (MSV) were less than the corresponding AVE values in the other constructs. Likewise, as noted in Table 2, except for SN, the square roots of the AVE values in the other five constructs were all greater than the rest of the inter-construct correlations. This finding indicated that the proposed model achieved discriminant validity on five constructs, except for SN. The inadequacy of the discriminant validity of SN was identified as a limitation of the current study.

### 4.2. SEM Results

**Figure 3:**
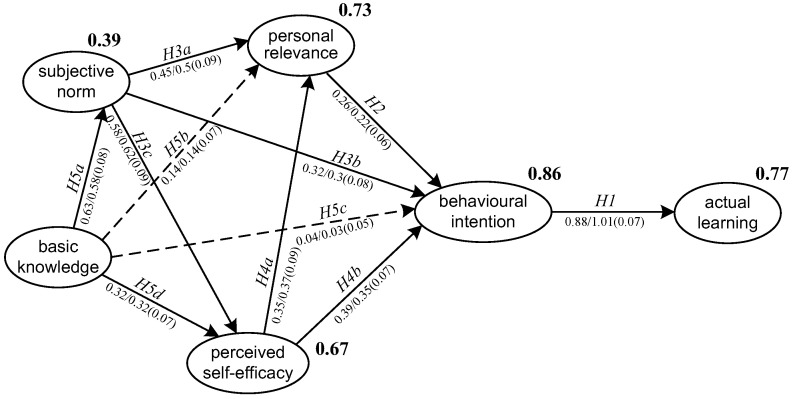
SEM results of the proposed structural model.

SEM was conducted to test the hypotheses proposed in relation to the model and the results demonstrated a good model fit (*χ*^2^ = 537.642, *df* = 219, *χ*^2^/*df* = 2.455, *p* < 0.001, CFI = 0.942, TLI = 0.933, RMSEA = 0.083, and SRMR = 0.056). Figure 3 reported the standardized and unstandardized estimates of the path coefficients in the model. The squared multiple correlations (*R*^2^) of the endogenous variables which estimated the percentage of variance explained were also reported. The variance explained among the indicator variables were at a medium to high level, except for SN, whose *R*^2^ is 0.39. This indicated that SN might be better explained by some other factors or by BKn combined with other factors.

In Figure 3, the numbers before and after the slashes are standardized and unstandardized estimates of the path coefficients, respectively. Standardized error is in parentheses. All significant paths (significant at the 0.001 level) are presented in solid lines, and non-significant paths in dashed lines. The squared multiple correlations (*R*^2^) of the endogenous variables were labelled in bold to the top right of each endogenous variable.

As shown in Table 4, nine out of the eleven hypotheses were supported. First, BI had a significantly strong and positive impact on AL (*β* = 0.88, *p* < 0.001) and explained 77 percent of the variance in AL. Thus, H1 was well supported. Second, the theoretical hypotheses in relation to the TPB were well confirmed in this study. BI was significantly predicted by PR (*β* = 0.26, *p* < 0.001), SN (*β* = 0.32, *p* < 0.001) and PSE (*β* = 0.39, *p* < 0.001). The total variance explained jointly by the three factors was 86 percent. Conversely, BKn did not have a direct effect on BI (*β* = 0.04, *p* = 0.489). Thus, H2, H3b and H4b were supported but H5c was not supported. Third, PR was significantly and positively predicted by SN (*β* = 0.45, *p* < 0.001) and PSE (*β* = 0.35, *p* < 0.001), whereas BKn failed to predict PR (*β* = 0.14, *p* = 0.029). Accordingly, H3a and H4a were supported, but H5b was not supported. Fourth, both SN (*β* = 0.58, *p* < 0.001) and BKn (*β* = 0.32, *p* < 0.001) had significant and positive impact on PSE, and jointly explained 67 percent of the total variance in PSE. In addition, BKn had a significantly positive effect on SN (*β* = 0.63, *p* < 0.001) and explained 39 percent of its variance. Thus, H3c, H5d and H5a were all supported in the current study.

## 5. Discussion

Understanding people’s responses to emerging technologies is a prerequisite for implementing effective interventions designed to facilitate behavioural changes that are needed to meet the demands of a high-tech society [16]. Whilst AI is increasingly and widely utilized in the medical practice, medical students’ behavioural intention to learn AI has rarely been studied [72]. Given the possibility that AI-based applications will acquire many of their roles and engender new tasks in clinical care, medical students’ intention to learn AI must be extensively investigated. This TPB-based study surveyed senior undergraduates and postgraduates in a medical institution to learn AI and explored the structural relations among their behavioural, normative and control beliefs. Moreover, participants’ medical AI literacy was also taken into consideration as a background factor due to its significance to the TPB model [30,32,65]. Contextualizing the TPB-based beliefs into learning medical AI, the current study chose personal relevance as their attitude toward the behaviour, self-efficacy as their perceived behavioural control. The participants’ actual learning of medical AI was also involved in the structural model proposed to confirm the reliability of behavioural intention as a significant indicator of actual behaviour. The results from 211 medical students (senior year undergraduates and master and doctoral postgraduates) in a medical university on the Chinese mainland showed that personal relevance of medical AI, subjective norms related to and perceived self-efficacy of learning medical AI could directly and positively predict the medical students’ intention to learn AI, which in turn drastically determined their actual behaviour of learning medical AI. Furthermore, participants’ basic knowledge of medical AI only had indirect effect on their intention. The findings in this study generally echoed what was found in previous studies that investigated AI learning based on the TPB [30,32,65], establishing validity of the TPB for identifying factors that influence behavioural intention toward learning AI in the context of medical education.

The current study proposed a validated six-construct model based on the TPB through CFA and SEM. This model could be used to measure students’ intention to learn AI in the medical context. Given the limited number of studies in relation to learning medical AI [10,11], this study could enrich the applicability of TPB in the medical education context. For medical students, their experience of learning AI technologies for medical care may contribute to preparing them for AI-enhanced clinical practice and enhance their intention toward learning medical AI. Our findings suggested that to foster strong intention to learn medical AI, course teachers and policy makers in medical institutions need to raise students’ personal relevance of medical AI, enhance their subjective norms related to and the perceived self-efficacy of learning medical AI. Meanwhile, exposing the participants to medical AI technologies may directly strengthen and reinforce their behavioural intention in question. In turn, their intention toward AI may positively reinforce their actual learning behaviour.

Personal relevance of medical AI reflected the participants’ attitude toward the behaviour of learning medical AI, and it had a significantly positive effect on their intention. The medical students’ exposure to AI technologies reflects a positive attitude toward medical AI by ascertaining that the adoption of medical AI increases one’s productivity and performance in clinical practice. This finding suggested that raising the personal relevance of AI in real-world situations might motivate the students to learn medical AI. Those who consider AI technologies to be a more relevant means of increasing their productivity in clinical practice may be more engaged in the actual learning of medical AI. Pedagogically, course teachers and mentors may use more clinical cases that involved medical AI to illustrate that AI-based applications can achieve higher quality practice such as precision medicine [73]. Conversely, subjective norms and self-efficacy significantly predicted personal relevance, indicating that apart from their respective direct effects, normative and control beliefs had indirect effects on intention through attitudinal variables. The findings are congruent with what was found in previous TPB studies [46,47,53,55].

Subjective norms related to learning AI were found to have both direct and indirect effect on medical students’ intention toward learning AI, which corroborated the findings in previous studies based on the TPB [45,46,52]. Particularly, this study revealed that the indirect effects of subjective norm (normative belief) on intention were medicated by personal relevance (behavioural belief) and self-efficacy (control belief). As such, establishing a supportive subjective norm may make medical students more aware of the significance of AI technologies used in medicine and grow more confident in learning those technologies for medical practice, and thus they may foster stronger intention of learning medical AI. On the other hand, apart from the direct and indirect effects that subjective norms had on intention, this study also found that subjective norms medicated the effect of basic knowledge on intention. This may indicate that participants having better knowledge of medical AI may witness a more supportive subjective norm and thus develop a stronger intention to learn medical AI. Such a finding echoed recent studies pertaining to the TPB [30,65] and lent support to Fishbein and Ajzen’s model [43] that depicted knowledge as a key determinant in the TPB.

Consistent with previous TPB studies [22,30,33], the current study revealed that perceived self-efficacy was the most crucial factor which had a significantly direct effect on participants’ intention toward learning medical AI. Meanwhile, self-efficacy was found to have a significant and indirect effect on participants’ intention through personal relevance. This finding was congruent with previous TPB studies regarding technology acceptance [55,56]. Conversely, it was found in this study that the indirect effect of basic knowledge on intention could not be medicated by personal relevance. To some extent, those results lent some support to Ajzen’s postulation that “when knowledge about actual behavioural control is limited, perceived behavioural control can be used as a proxy to aid in the prediction of behaviour” [16] (p. 316). In view of the relationship between perceived behavioural control and attitude toward the behaviour, this study echoed Chai et al.’s finding [30] that perceived self-efficacy could predict attitudinal factors of intention. On the other hand, this study also confirmed that vicarious experience and verbal messages could shape self-efficacy as its two major sources because basic knowledge and subjective norms were found to be two determinants of self-efficacy. Therefore, in our context, addressing medical students’ self-efficacy was considered a key route to enhancing their intention of learning medical AI. In turn, increasing their knowledge about medical AI and exposing them to a favourable subjective norm related to medical AI could augment their self-efficacy of learning AI for medical use.

Basic knowledge of medical AI was integrated into the proposed model based on Fishbein and Ajzen’s work [43] that depicted knowledge or information as a determinant of both attitude toward the behaviour and perceived behavioural control. However, it was uncovered in this study that basic knowledge of medical AI did not predict behavioural intention directly. Its effect on intention was mediated by subjective norms or self-efficacy. This suggested that basic knowledge of medical AI was not a sufficient condition for the participants to develop an intention toward learning medical AI. Conversely, being medically AI literate can contribute to establishing a supportive subjective norm or growing more confident in learning medical AI, which may then foster a strong intention to learn medical AI. In Chai et al.’s study [30], it was also found that being AI literate was not a direct predictor of behavioural intention and students’ readiness to learn AI. On the other hand, this study found that basic knowledge failed to predict personal relevance, which was operationalized as the attitudinal factor in the TPB. This indicated that gaining knowledge of medical AI might not make the participants aware that medical AI could improve their productivity or performance. However, this study revealed that basic knowledge had an indirect effect on personal relevance through subjective norms, meaning that those who are medically AI literate might realize medical AI would enhance their productivity through a supportive subjective norm from important others rather than by themselves.

To conclude, the emergence of AI has greatly changed medical science and clinical practice, and as proclaimed by researchers [30,74,75], medical education must embrace AI-enhanced practice and reform itself accordingly. Medical students need to prepare themselves to learn AI technologies for clinical practice. Given that students will work in an AI-empowered society in the future [76], fuelling students’ intention toward learning AI is crucial. Based on the findings in this study, we suggest that medical institutions and the mentors should provide a supportive subjective norm and expose the students to knowledge of medical AI more frequently. By so doing, medical students may realize the relevance of medical AI to their career, grow more confident in learning AI and thus develop strong intention toward learning AI for medical practice.

Several limitations were identified in this study. First, the sample was limited to one medical university on the Chinese mainland. Future studies should base their surveys on a larger and richer sample of participants to ensure the representativeness. Second, the divergent validity of SN on the construct level needs to be improved so that the risk of validity threat to this study could be lowered to some extent. Third, as pinpointed by Ajzen [16], theoretically, perceived behavioural control moderates the effect of attitudes and subjective norm on behavioural intention, and future studies may test the moderating effect and seek out interaction evidence so that the understanding of the TPB could be deepened empirically [77]. Fourth, this study only investigated four factors that are assumed to influence BI. In the context of medical education, more factors such as medical AI for social good [30], ethical concerns of medical AI, perceived technological support, etc., might be integrated into the proposed model in the study for examining the structural relationships in the future.

## 6. Conclusions and Implications

The present study investigated medical students’ perceptions of and behavioural intention toward learning AI technologies for medicine. The proposed model was based on the TPB and was composed of six constructs, namely, (1) BKn of medical AI, (2) SN about learning medical AI, (3) PR of medial AI, (4) PSE of learning medical AI, (5) BI toward learning medical AI and (6) AL of medical AI. The model fit was psychometrically satisfactory, and the results showed that all the factors in the model contributed directly or indirectly to the participants’ BI toward learning medical AI. It was found that (1) PR, SN and PSE all contributed to the formation of BI toward learning medical AI; (2) BKn of medical AI could predict SN and PSE but failed to predict PR and BI directly; (3) PR mediated the effect of PSE on BI and the effect of SN on BI; and (4) BI was a good predictor of AL.

Theoretically, the implications of the present study are twofold: (1) medical students’ behavioural intention to learn medical AI was modelled by the TPB framework well, echoing Ajzen’s claim [16] that the framework has been adopted successfully to explain and predict behaviour in a multitude of behavioural domains; (2) the present study extended the TPB framework by integrating a cognitive construct (i.e., BKn) which was found to indirectly predict BI but directly predict SN and PSE. Expanding the TPB by integrating a technology literacy-related construct led to more insightful perspectives in interpreting medical students’ perception of medical AI. Future research may further confirm the effect of AI literacy on medical students’ behavioural intention to learn AI, as well as their actual learning of medical AI.

In practice, according to the findings of this study, knowledge of medical AI did not predict students’ behavioural intention to learn medical AI, indicating that focusing solely on teaching medical knowledge may not directly promote medical students’ intention to learn and use medical AI technologies. Rather, to raise their behavioural intention to learn medical AI, practitioners and researchers in medical education need to help students realize that AI technology used for clinical practice is exceedingly relevant in their future career and demonstrate in their own clinical practice that medical AI is conducive to their practising medicine and conducting research as a doctor. Meanwhile, they need to help medical students foster stronger self-efficacy in using medical AI in their clinical practice. To that end, teachers in medical education should build up a learning environment where medical AI technologies are no longer just technologies read by students in textbooks but can play a pivotal role in clinical cases shared by their professors and course teachers.

## Figures and Tables

**Table 1:** CFA results (*n* = 211).

Measure	Item	Mean	SD	Standardized Estimate	*t*-Value
PR	PR1	4.18	1.13	0.95	--
	PR2	4.20	1.13	0.98	38.31 **
	PR3	4.27	1.12	0.98	36.21 **
	PR4	4.29	1.11	0.90	24.89 **
SN	SN1	3.61	1.39	0.66	--
	SN2	4.11	1.21	0.80	9.91 **
	SN3	4.14	1.25	0.80	9.91 **
	SN4	4.49	1.10	0.83	10.28 **
PSE	PSE1	4.01	1.22	0.85	--
	PSE2	4.22	1.14	0.94	19.08 **
	PSE3	4.37	1.09	0.92	18.47 **
BKn	BKn1	3.02	1.39	0.74	--
	BKn2	3.37	1.32	0.88	12.66 **
	BKn3	3.96	1.28	0.76	10.95 **
	BKn4	3.72	1.32	0.80	11.55 **
BI	BI1	4.48	1.05	0.88	--
	BI2	4.47	1.03	0.89	18.93 **
	BI3	4.55	1.03	0.91	20.02 **
	BI4	4.32	1.13	0.91	19.76 **
AL	AL1	3.91	1.22	0.87	--
	AL2	3.79	1.26	0.80	15.00 **
	AL3	4.11	1.11	0.93	20.06 **
	AL4	4.03	1.16	0.91	19.11 **

PR = personal relevance, SN = subjective norm, PSE = perceived self-efficacy, BKn = basic knowledge, BI = behavioral intention, AL = actual learning; ** *p* < 0.001.

**Table 2:** Correlation matrix and descriptive statistics on the construct level.

	1	2	3	4	5	6
1. PR	(0.95)					
2. SN	0.81 **	(0.77)				
3. PSE	0.79 **	0.78 **	(0.90)			
4. BKn	0.66 **	0.63 **	0.68 **	(0.80)		
5. BI	0.84 **	0.85 **	0.85 **	0.64 **	(0.90)	
6. AL	0.76 **	0.78 **	0.85 **	0.75 **	0.85 **	(0.88)
Mean	4.24	4.09	4.20	3.52	4.46	3.96
SD	1.08	1.03	1.07	1.13	0.98	1.08
Skewness	−0.91	−0.44	−0.88	−0.06	−1.07	−0.48
Kurtosis	1.14	0.33	1.18	−0.28	2.39	0.02
Cronbach α	0.98	0.85	0.93	0.87	0.94	0.93

PR = personal relevance, SN = subjective norm, PSE = perceived self-efficacy, BKn = basic knowledge, BI = behavioral intention, AL = actual learning; The square root of average variance extracted is in parentheses on the diagonal; ** *p* < 0.001.

**Table 3:** Reliability and validity results.

Measure	CR	AVE	MSV	MaxR(H)
PR	0.98	0.91	0.71	0.99
SN	0.86	0.60	0.72	0.87
PSE	0.93	0.82	0.72	0.94
BKn	0.88	0.64	0.56	0.89
BI	0.94	0.81	0.73	0.94
AL	0.93	0.77	0.73	0.94

CR = composite reliability; AVE = average variance extracted, MSV = maximum shared variance, MaxR(H) = McDonald’s construct reliability; PR = personal relevance, SN = subjective norm, PSE = perceived self-efficacy, BKn = basic knowledge, BI = behavioral intention, AL = actual learning.

**Table 4:** SEM results (*n* = 211).

Hypothesis	Path	*β*-Value	*Β*-Value	*SE*	*t*-Value	Result
H1	BI → AL	0.88	1.01	0.07	14.33 **	Supported
H2	PR → BI	0.26	0.22	0.06	3.60 **	Supported
H3a	SN → PR	0.45	0.50	0.09	5.40 **	Supported
H3b	SN → BI	0.32	0.30	0.08	4.06 **	Supported
H3c	SN → PSE	0.58	0.62	0.09	7.22 **	Supported
H4a	PSE → PR	0.35	0.37	0.09	4.29 **	Supported
H4b	PSE → BI	0.39	0.35	0.07	5.38 **	Supported
H5a	BKn → SN	0.63	0.58	0.08	7.61 **	Supported
H5b	BKn → PR	0.14	0.14	0.07	2.18	Not supported
H5c	BKn → BI	0.04	0.03	0.05	0.69	Not supported
H5d	BKn → PSE	0.32	0.32	0.07	4.35 **	Supported

*SE* = standardized error; PR = personal relevance, SN = subjective norm, PSE = perceived self-efficacy, BKn = basic knowledge, BI = behavioral intention, AL = actual learning; ** *p* < 0.001.

## Data Availability

The data presented in this study are available on request from the corresponding author.

## Appendix A

Questionnaire items (finalized)

PR1. Using medical AI technology enables me to accomplish clinical tasks more quickly.
PR2. Using medical AI technology improves my clinical performance.
PR3. Using medical AI technology increases my clinical productivity.
PR4. Using medical AI technology enhances my effectiveness.
SN1. My school organizes enrichment lessons for us to learn more about medical AI technologies.
SN2. My peers and/or parents encourage me to participate in innovative medical AI learning activities.
SN3. My mentors/boss have emphasized the necessity to work creatively using medical AI technology.
SN4. My classmates feel that it is necessary to learn how to work with medical AI technology.
SE1. I am certain I can understand the most difficult materials presented in the courses about medical AI.
SE2. I feel confident that I will do well in clinical practice involving medical AI.
SE3. I am confident I can learn the basic concepts taught in the courses about medical AI.
BKn1. I understand how computers process medical imaging to produce visual recognition and analysis.
BKn2. I understand how AI technology optimizes the health care solutions.
BKn3. I understand why AI-assisted genomic diagnostics needs big data for machine learning.
BKn4. I understand how AI assistant in online patient guidance system handle human-computer interaction.
BI1. I will continue to learn about medical AI technology in the future.
BI2. I will pay attention to emerging AI applications used in medical practice.
BI3. I expect that I would be concerned about medical AI development in the future.
BI4. I plan to spend time in learning medical AI technology in the future.
AL1. I have intentionally searched and viewed educational videos about medical AI.
AL2. I have interacted with medical AI applications to understand how they work.
AL3. I have studied about medical AI through books and journals.
AL4. I have attended lessons about medical AI in schools or outside schools.
